# A positive feedback loop of the TAZ/β-catenin axis promotes *Helicobacter pylori*-associated gastric carcinogenesis

**DOI:** 10.3389/fmicb.2022.1065462

**Published:** 2022-12-23

**Authors:** Xinbo Xu, Chunxi Shu, Xidong Wu, Yaobin Ouyang, Hong Cheng, Yanan Zhou, Huan Wang, Cong He, Chuan Xie, Xingxing He, Junbo Hong, Nonghua Lu, Zhongming Ge, Yin Zhu, Nianshuang Li

**Affiliations:** ^1^Digestive Disease Hospital, The First Affiliated Hospital of Nanchang University, Nanchang, China; ^2^Department of Gastroenterology, The First Affiliated Hospital of Nanchang University, Nanchang, China; ^3^Department of Drug Safety Evaluation, Jiangxi Testing Center of Medical Instruments, Nanchang, China; ^4^Jiangxi Institute of Digestive Disease, The First Affiliated Hospital of Nanchang University, Nanchang, China; ^5^Division of Comparative Medicine, Massachusetts Institute of Technology, Cambridge, MA, United States

**Keywords:** *Helicobacter Pylori*, CagA, TAZ, β-catenin, gastric carcinogenesis

## Abstract

**Background:**

*Helicobacter pylori* infection is the strongest known risk factor for gastric cancer. The Hippo signaling pathway controls organ size and maintains tissue homeostasis by coordinately regulating cell growth and proliferation. Here, we demonstrate the interactive role of TAZ, the transcriptional coactivator of the Hippo pathway, and beta-catenin in promoting the pathogenesis of *H. pylori* infection.

**Methods:**

TAZ expression was evaluated in human gastric tissues and *H. pylori*-infected insulin–gastrin (INS-GAS) mice. Western blot, immunofluorescence, immunohistochemistry, and RT–PCR assays were performed. Coimmunoprecipitation was performed to examine the interaction between TAZ and β-catenin. TAZ and β-catenin were silenced using small interfering RNAs. HA-β-catenin and Flag-TAZ were constructed.

**Results:**

Increased TAZ was noted in human gastric cancer tissues compared to chronic gastritis tissues and in *H. pylori*-positive gastritis tissues compared to *H. pylori*-negative gastritis tissues. In addition, *H. pylori* infection induced TAZ expression and nuclear accumulation in the gastric tissue of INS-GAS mice and cultured gastric epithelial cells, which was dependent on the virulence factor CagA. Moreover, TAZ or β-catenin knockdown significantly suppressed *H. pylori* infection-induced cell growth, survival, and invasion. Furthermore, the interactive regulation of TAZ and β-catenin activation was revealed. Finally, β-catenin was required for *H. pylori*-induced TAZ activation.

**Conclusion:**

These findings suggest the existence of a positive feedback loop of activation between TAZ and β-catenin that could play an important role in CagA+ *H. pylori* infection-induced gastric carcinogenesis. TAZ inhibition represents a potential target for the prevention of *H. pylori* infection-associated gastric cancer.

## Background

*Helicobacter pylori* infection is the strongest known risk factor for the development of gastric cancer and the fourth leading cause of cancer death worldwide, particularly in East Asian populations Approximately half of the world’s population is infected with *H. pylori* (Sung et al., 2021). *H. pylori* infection can cause chronic active gastritis that can progress through Correa’s cascade to intestinal metaplasia, dysplasia, and finally gastric adenocarcinoma (Correa and Piazuelo, 2011). CagA is the most well-characterized virulence factor that links *H. pylori* infection to gastric carcinogenesis (Ansari and Yamaoka, 2019). Patients infected with CagA-positive *H. pylori* strains are suggested to have an increased risk of gastric carcinoma compared to CagA-negative subjects (Park et al., 2018). Accumulating data suggest that the interaction between bacterial virulence factors and host gastric epithelial cells induces aberrant activation of multiple oncogenic signaling pathways (PI3K/AKT, Wnt/β-catenin, etc) and subsequently results in gastric carcinogenesis (Xie et al., 2018; Cao et al., 2022). Another bacterial virulence factor, VacA, is also linked to clinical phenotypes, such as cellular vacuolation of *H. pylori* infection. Numerous studies have shown that eradication of *H. pylori* based on regiments including systematic PPI and antibiotics could significantly reduce gastric cancer risk before the development of atrophic gastritis and intestinal metaplasia (Liou et al., 2020; Yan et al., 2022). However, it remains unclear whether *H. pylori* eradication therapy reduces the incidence of gastric cancer once premalignant gastric lesions develop. Therefore, the identification of novel and reliable biomarkers is essential for the prediction and prevention of gastric cancer associated with *H. pylori* infection.

The Hippo signaling pathway maintains organ size and tissue homeostasis *via* the regulation of cell proliferation, survival, and differentiation (Russell and Camargo, 2022). YAP and its paralogue TAZ (also known as WWTR1) act as transcriptional coactivators and effectors of the Hippo signaling cascade. Activation of the upstream kinases LATS1/2 and MST1/2 leads to the phosphorylation and inactivation of YAP and TAZ due to cytoplasmic retention and degradation (Totaro et al., 2018; Mohajan et al., 2021). Inhibition of the Hippo kinase cascade increases the nuclear localization of YAP and TAZ, which are responsible for the activation of downstream genes, such as *CTGF*, *CYR61,* and *c-MYC* (Pobbati and Hong, 2020). It is increasingly evident that the dysregulation of the Hippo signaling pathway plays a vital role in *H. pylori* infection-induced gastric tumorigenesis (Molina-Castro et al., 2020). We previously reported that *H. pylori* infection promotes total YAP levels and nuclear localization, which is also dependent on virulence CagA. As a result, neoplastic transformation is initiated in gastric epithelial cells through the epithelial-mesenchymal transition (EMT), a process by which epithelial cells lose their cell polarity and cell–cell adhesion and subsequently acquire migratory and invasive characteristics (Yang et al., 2020). We also found higher TAZ expression levels in gastric carcinoma tissues compared with adjacent normal tissues (Li et al., 2018). However, the role of YAP paralogue TAZ in the pathogenesis of *H. pylori* infection has not been explored.

Independent of the Hippo cascade, β-catenin is the core effector of the canonical Wnt signaling pathway (Liu et al., 2022). The nuclear accumulation of β-catenin upon the activation of the Wnt pathway accounts for the upregulation of target genes such as *c-MYC* and *cyclin D1* (Zhang and Wang, 2020). Accumulating data suggest that the Hippo and Wnt signaling pathways regulate overlapping biological processes, including tissue growth, development, and homeostasis (Sylvester and Colnot, 2014; Li et al., 2019). TAZ is defined as the downstream element of the Wnt/β-catenin signaling cascade. β-catenin phosphorylation leads to TAZ degradation by bridging TAZ to the E3 ubiquitin ligase β-TrCP. In Wnt-ON cells, β-catenin dissociation from the destruction complex impairs TAZ degradation, allowing the nuclear accumulation of TAZ and β-catenin (Azzolin et al., 2012). It has been reported that infection with carcinogenic *H. pylori* strains induces nuclear translocation of β-catenin in a rodent model and gastric cells (Franco et al., 2005). It remains unclear whether the intersection of TAZ and the β-catenin pathway is implicated in *H. pylori*-mediated carcinogenesis.

In the present study, we elucidated a mechanism by which the activation of TAZ promotes the β-catenin pathway to trigger gastric epithelial malignant transformation in response to *H. pylori* infection. Here, we showed that *H. pylori* strains expressing high levels of CagA significantly upregulated TAZ expression and nuclear accumulation in gastric epithelial cells and transgenic INS-GAS mice. TAZ knockdown reduced *H. pylori* infection-induced expression, nuclear translocation and transcriptional activity of β-catenin. Interestingly, TAZ and β-catenin were mainly located in the cellular cytoplasm and plasma membrane in human specimens with chronic gastritis, whereas partial nuclear colocalization of TAZ with β-catenin was observed in human gastric cancer tissues. *In vitro* studies revealed that *H. pylori* infection augmented the direct interaction of TAZ with β-catenin *via* the CagA-dependent mechanism. Furthermore, β-catenin knockdown significantly suppressed the activation of TAZ and its downstream genes following *H. pylori* infection. Moreover, TAZ knockdown by siRNA reduced *H. pylori* infection-triggered neoplastic transformation including cell viability, proliferation, and invasion. These findings suggest that aberrant activation of TAZ is a marker of gastric carcinoma risk and that the feedback loop of the TAZ/β-catenin axis plays a crucial role in *H. pylori* infection-induced carcinogenesis.

## Materials and methods

### Cell culture and reagents

Human gastric epithelial cell lines, HFE-145 (immortalized, non-cancerous) and AGS (human diffuse type of gastric cancer) were cultured in DMEM and DMEM/F12, containing 10% FBS (Gibco), 100 U/ml penicillin, and 100 μg/ml streptomycin at 37°C in an atmosphere of 5% CO2.

### *Helicobacter pylori* strains

*CagA* + *VacA+ H. pylori* strain PMSS1 (pre-mouse Sydney strain 1) and its isogenic *cagA* mutant were established in our previous research. *CagA* + *VacA + H. pylori* strains 7.13 and an isogenic cagA were also included in this study, which were kindly provided by Dr. Richard Peek Jr. from the Vanderbilt Digestive Disease Research Center. All *H. pylori* strains were cultured on Campylobacter agar plates containing 10% sheep serum at 37°C under microaerophilic conditions. The measure methods of *H. pylori* bacterial density were as same as the previous study (Hu et al., 2019). Gastric epithelial cells were cocultured with *H. pylori* strains at MOI of 100.

### Human gastric specimens

A total of 40 human chronic non-atrophic gastritis (*n* = 20) and gastric carcinoma (*n* = 20) tissues were acquired from The First Affiliated Hospital of Nanchang University. Each group were divided into two subgroups: *H. pylori*-positive (*n* = 10) and *H. pylori*-negative (*n* = 10) individuals. The study protocol and exemption of informed consent were approved by the Ethics Committee of The First Affiliated Hospital of Nanchang University. Status of *H. pylori* infection for these clinical specimens was determined with a rapid urease test or Giemsa staining. Immunohistochemical staining was performed to examine expression profiles of TAZ on these samples, which were evaluated and scored for intensity (scaled 0–3) and frequency (scaled 0–4) by two pathologists blinded to sample identity. For statistical analysis, expression levels of TAZ proteins were illustrated by an expression score in range of 0 to 12 using the formula intensity × frequency (Li et al., 2018).

### Infection of mice with *Helicobacter pylori*

Animal care and experimental protocols were in accordance with guidelines established by the Institutional Animal Care and Use Committee of Nanchang University. The INS-GAS transgenic mice overexpressed pancreatic gastrin were purchased from Jackson Laboratory (Bar Harbor, ME). INS-GAS mice were orogastrically challenged with Brucella Broth (*n* = 6) or with 2 × 10^9^ CFU/ml *H. pylori* strain PMSS1 (*n* = 8) once every other day for a total of 5 times (Arnold et al., 2011; Li et al., 2020). Mice were euthanized at 4 months post infection, and gastric tissues were collected.

### Expression vectors, siRNAs, and transfection

The recombinant plasmids such as TAZ, β-catenin cDNAs were designed and synthesized by hitrobio (Beijing, China). The recombinant CagA plasmid was a generous gift from Prof. Shiming Yang, Third Military Medical University of China. Small interfering (si) RNA duplexes were obtained from GenePharma (Shanghai, China). Cells were transfected with appropriate plasmid or siRNA using Lipofectamine 3,000 (Thermo Scientific, Waltham, MA, United States) according to manufacturer’s instructions.

### Immunohistochemistry

Immunohistochemistry analysis was performed for gastric tissues from human and INS-GAS mice as previously described (22). These specimens were incubated with rabbit polyclonal anti-TAZ (Proteintech, Wuhan, China) at dilution of 1:400, followed by incubation with secondary antibody (PV6000, Zhongshan Golden-bridge, Beijing, China). Immunostained tissue slides were imaged on an upright confocal microscope (Nikon ECLIPSE Ni). Tissue sections were imaged at 200× and 400× magnification.

### Western blotting

Western blotting analysis was conducted as described previously (Xie et al., 2020). Primary anti-TAZ (#83669), anti-β-catenin (#37447), anti-β-tubulin (#2128), anti-Histone 3 (#4499), anti-GAPDH (#2118), anti-HA-Tag (#3724), anti-Myc (#9402), anti-Cyclin D1(#2978), anti-CYR61(#14479), anti-CTGF (#86641) antibodies were purchased from Cell Signal Technology (Beverly, MA, United States); anti-CagA (sc-28,368) were from Santa Cruz (Dallas, TX, United States), anti-β-actin (#20536-1-AP) from proteintech (Wuhan, China); anti-Flag (F1804) was purchased from Sigma-Aldrich. All primary antibodies were used at a dilution of 1:1000, except for the internal control antibodies including GAPDH and β-actin which were diluted at 1:2000. Briefly, the proteins were extracted after adding lysis buffer supplemented with protease inhibitors (Invitrogen, GA, United States). Equal amounts of the sample proteins were separated on SDS-PAGE gels and transferred to nitrocellulose membranes. After blocking with 5% milk, the membranes were incubated with the primary antibodies overnight at 4°C, and then incubated with HRP-conjugated secondary antibodies (Invitrogen, GA, United States) for 1 h at room temperature. The protein bands were visualized using Super Signal West Pico stable peroxide solution (Thermo Scientific) and collected using iBright imaging system (Thermo Scientific). β-actin and GAPDH were used as internal control to normalize protein expression in cells sample and animal tissues sample, respectively.

### Quantitative real-time PCR analysis

The qRT-PCR analysis was performed as described in our previous studies (Xie et al., 2020). Briefly, total RNA was extracted using TRIzol reagent (Invitrogen, GA, United States). The primer sequences are presented in Supplementary Table S1. The qPCR assays were performed with a QuantStudio 5 Real-time PCR system (Life Technologies) according to the manufacturer’s protocol. The GAPDH gene was used as an internal control.

### Luciferase assay

The TCF/LEF reported plasmid for β-catenin activity (M50 Super 8 × TOPFlash; plasmid#12456; Addgene, Cambridge, MA, United States) was a gift from Randall Moon (Veeman et al., 2003). For dual luciferase assay, cells were seeded into 12-well plates. The cells were transfected with 8 × TOPFlash plasmid and indicated siRNA. After transfection for 48 h, cells were co-cultured with *H. pylori* strain. The cell lysates were collected and subjected to a dual luciferase assay system (Yeasen Biotech, Shanghai, China) according to the manufacturer’s protocol.

### Immunofluorescence

For immunofluorescence staining, cells were washed with ice-cold PBS and fixed with 4% formaldehyde in PBS for 15 min. Then, the cells were permeabilized with 0.5% Triton X-100 for 10 min at room temperature. After blocking with 3% BSA for 30 min, the cells were incubated with primary antibodies against TAZ (dilution 1:100) or β-catenin (dilution 1:200) overnight at 4°C, and then incubated with Secondary Antibody (Alexa Fluor Plus 488 and Alexa Fluor Plus 555; Thermo Scientific). Cell nuclei were counters with DAPI. All images were acquired using a confocal fluorescence microscope (Leica Stellaris).

### Immunoprecipitation

For immunoprecipitation assay, cells were transfected with the indicated plasmids, and then collected with lysis buffer. In brief, the cell lysates were incubated overnight with the mixture of 1 ug of antibodies and beads at 4°C. Then the beads were washed three times with 1 ml of lysis buffer and then boiled in loading buffer. The samples were subjected to western blot analysis as described above.

### CCK8 and EDU assays

Cells were transfected with indicated plasmid vector or siRNA, and then seeded into 96-well plates at a density of 2 × 10^3^ cells/100 μl per well. The cells were infected with *H. pylori* strain at an MOI of 50 for the indicated time. The CCK-8 assay (TransGen Biotech, Beijing, China) was performed according to the manufacturer’s instructions. The optical density values were measured at a wavelength of 450 nm using a Molecular Devices SpectraMax M2e. For EDU assay, the relative viability of cells was determined by Cell-Light EDU Apollo 488 *in Vitro* imaging kit (RiboBio) following the kit protocol. All images were acquired and quantified using the high-content screening platform In-Cell Analyzer 2,200 (GE Healthcare).

### Boyden chamber assay

Boyden chamber assay is useful tool to study cell migration and invasion. For cell invasion detection, about 2 × 10^5^ cells were plated into the upper Boyden chamber (8 μm pore size, Corning, NY, United States) with Matrigel-precoated inserts (BD Science, United States). For cell migration detection, Matrigel was not required. DMEM/F12 medium supplemented with 20% FBS was added in the lower chamber. After transfection and *H. pylori* infection, Cells adhering to the lower surface were fixed with methanol for 30 min, stained with 1% crystal violet for 15 min. The cells on the upper surface of the filters were gently wiped and counted under the inverted microscope (Nikon, ECLIPSE Ti).

### Statistical analyses

All the statistical analysis was performed using SPSS 21.0 software. Data are presented as mean ± SD of three independent experiments. Studies for continuous variables were statistically analyzed using Student’s *t*-test or One-way ANOVA. The immunohistochemical data from human clinical specimens was statistically analyzed using Mann–Whitney test. The results were considered statistically significant at *p* < 0.05 (***, *p* < 0.001; **, *p* < 0.01; *, *p* < 0.05).

## Results

### Expression of gastric TAZ was elevated in *Helicobacter pylori* + human gastritis tissues and *Helicobacter pylori*-infected INS-GAS mice

**Figure 1:**
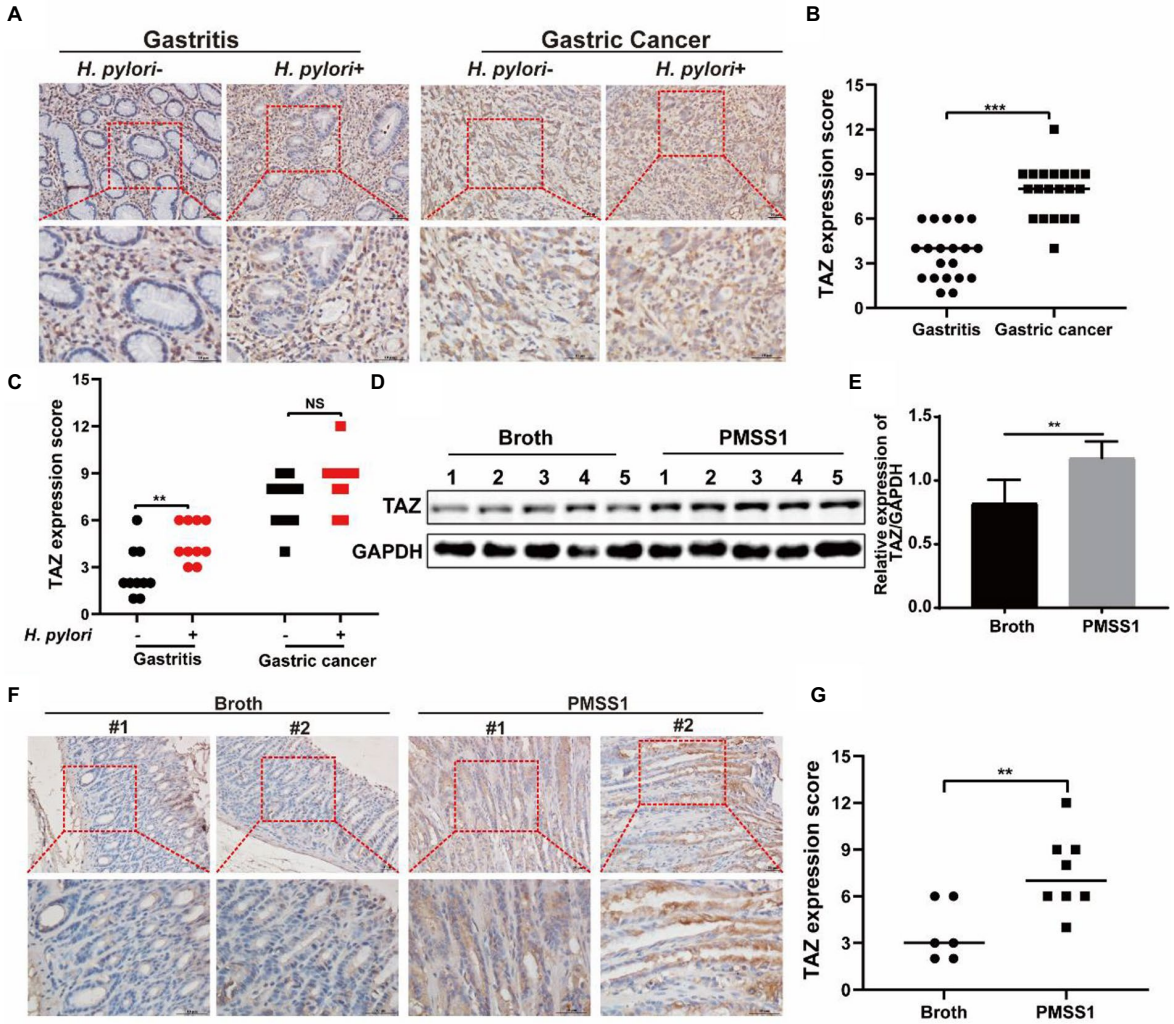
TAZ expression levels in human gastric tissues and INS-GAS mice infected with *H. pylori* PMSS1. **(A)** Immunohistochemistry for TAZ expression in human chronic gastritis and gastric cancer tissues. Representative images of TAZ (magnification 200× and 400×, scale bars =50 μm). **(B)** Quantitative analysis of TAZ expression in all cell types in gastritis and gastric cancer tissues from both *H. pylori*-negative and *H. pylori*-positive subjects. **(C)** Quantitative immunohistochemistry analysis of TAZ levels in *H. pylori*-positive or *H. pylori*-negative gastric tissues. **(D)** Western blot of TAZ protein expression in gastric tissues of INS-GAS mice infected with *H. pylori* PMSS1 strain or Brucella broth treatment. **(E)** Statistical analysis of the intensity of western blot bands. **(F)** Immunohistochemistry staining for TAZ expression in the *H. pylori*-infected INS-GAS mice. Representative images of TAZ (magnification 200× and 400×, scale bars =50 μm). **(G)** Quantitative analysis of TAZ levels. **p < 0.01, ***p < 0.001, *NS*, *not significant.* Scare bars in **(A,E)**, 10 μm.

We previously reported augmented YAP expression in preneoplastic lesions of human gastric tissues (Li et al., 2018). In this study, we first examined the expression patterns of the YAP homologue TAZ in clinical specimens. As shown in Figures 1A,B, the expression of gastric TAZ was strongly increased in human gastric cancer tissues compared to that observed in chronic nonatrophic gastritis tissues. *H. pylori* infection is linked to the initiation of chronic active gastritis and significantly increases the risk of gastric adenocarcinoma (Crowe, 2019). Therefore, the TAZ expression levels were further compared between *H. pylori*-positive and *H. pylori*-negative subjects. Interestingly, *H. pylori*-positive gastritis tissues tended to have higher levels of TAZ than *H. pylori*-negative gastritis tissues. However, no significant difference in TAZ expression was noted between *H. pylori*-infected and -uninfected gastric cancer individuals (Figures 1A,C). Next, we utilized INS-GAS mice, which overexpress human pancreatic gastrin and have been widely used for studying the pathogenesis of *H. pylori* infection (14), to characterize the effects of *H. pylori* on TAZ. Western blot analysis showed that gastric TAZ expression was significantly increased in *H. pylori*-infected mice compared with sham controls (Figures 1D,E). In addition, a significant increase in gastric TAZ expression was noted in *H. pylori*-infected mice compared to uninfected animals based on immunohistochemistry analysis (Figure 1F) and quantitative image analysis (Figure 1G). These results indicated that *H. pylori* infection triggers TAZ activation, which may be implicated in the initiation of gastric neoplastic lesions.

### *Helicobacter pylori* infection led to TAZ upregulation and nuclear accumulation in a CagA-dependent manner

**Figure 2:**
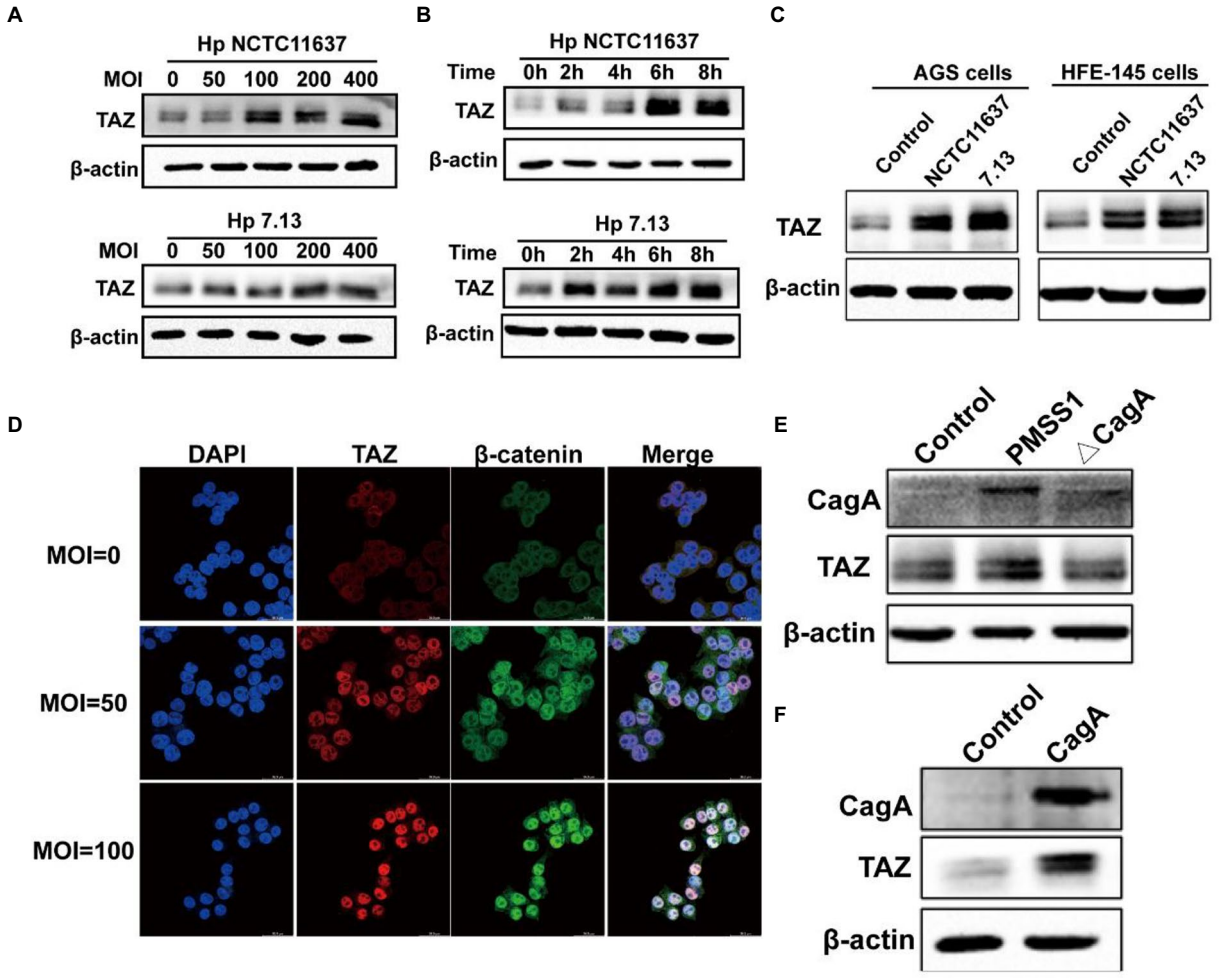
*Helicobacter pylori* infection promoted TAZ expression and nuclear localization. **(A)** Western blot for TAZ protein levels in human gastric epithelial cells infected with *H. pylori* NCTC11637 or 7.13 at different MOI for 6 h. **(B)** Western blot for TAZ levels in AGS cells treated with *H. pylori* strains at different time points at MOI of 100. **(C)** Western blot for TAZ levels in different gastric epithelial cells (AGS and HFE145) following infection with different *H. pylori* strains at MOI of 100 for 6 h. **(D)** Immunofluorescence staining for TAZ and β-catenin cellular localization after *H. pylori* NCTC11637 infection at different MOI for 6 h. (Magnification 400×, scale bars =30 μm) **(E)** Western blot for TAZ expression in gastric epithelial cells infected with PMSS1 or its isogenic CagA- mutant. **(F)** Western blot for TAZ expression in gastric cells after transfection with the CagA expression vector.

To recapitulate the effects of *H. pylori* infection on gastric TAZ expression in human gastric tissues and INS-GAS mice, human gastric epithelial AGS cells were cocultured with the CagA+ *H. pylori* strain NCTC11637 or 7.13. *H. pylori* infection significantly increased TAZ protein expression in both an MOI-dependent (50, 100, 200 and 400 MOI) (Figure 2A) and a time-dependent manner (2 h, 4 h, 6 h, and 8 h) (Figure 2B). Similarly, an increase was noted in total TAZ expression in normal human gastric epithelial HFE145 cells infected with *H. pylori* (Figure 2C). In addition, the intracellular localization of TAZ was increased after treatment with *H. pylori* strains, as shown by immunofluorescence staining. Importantly, the increase of *H. pylori* MOIs was correlated with the promotion of TAZ translocation from the cytoplasm to the nucleus (Figure 2D). These *in vitro* data clearly indicated that *H. pylori* infection leads to TAZ upregulation and activation.

CagA, which is delivered into gastric epithelial cells *via* the type IV secretion system (T4SS), has been considered an oncoprotein that induces gastric carcinogenesis (30). To determine the role of CagA in *H. pylori*-induced activation of TAZ, AGS cells were cultured alone or cocultured with *H. pylori* PMSS1 wild-type or its CagA-deficient isogenic mutant strain. We found that CagA knockout significantly inhibited the induction of TAZ in response to *H. pylori* infection (Figure 2E). Furthermore, transient overexpression of CagA *via* plasmid transfection into AGS cells strongly increased TAZ expression (Figure 2F). Furthermore, the recombinant CagA overexpression caused an marked increase in the nuclear localization of TAZ. These findings suggest that bacterial CagA is integral for *H. pylori*-induced TAZ activation.

### TAZ knockdown significantly inhibited *Helicobacter pylori* infection-induced gastric epithelial cell malignant transformation

**Figure 3:**
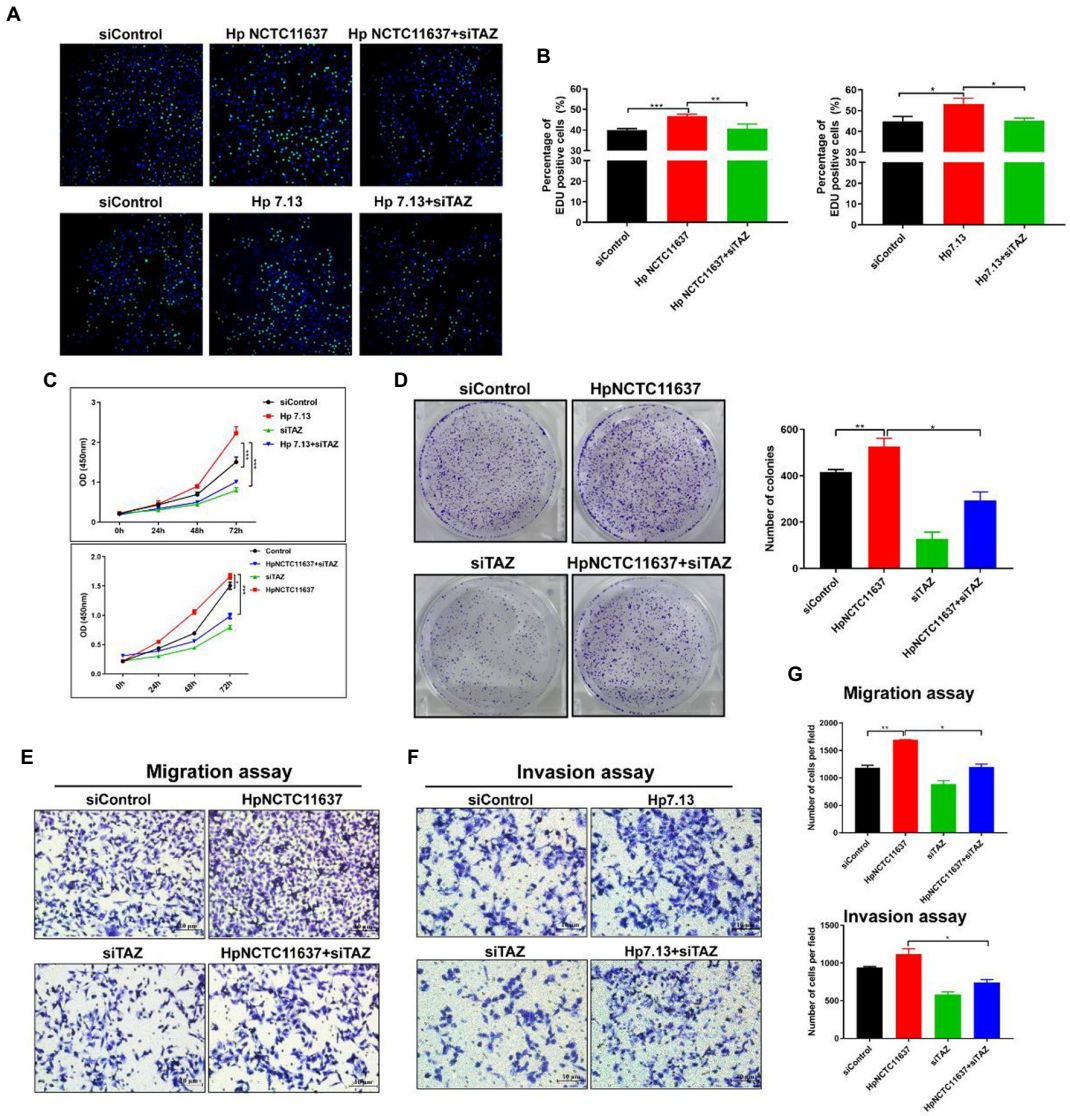
TAZ knockdown significantly inhibited *H. pylori* infection-induced cell proliferation, invasion and migration. **(A)** EdU assay for cell proliferation in AGS cells following infection with *H. pylori* (MOI = 100) and transfection with TAZ siRNA. Representative confocal images of EdU staining. **(B)** Quantification of the percentage of EdU-positive cells. **(C)** CCK8 viability assay for cell proliferation in AGS cells treated with *H. pylori* alone or in combination with TAZ siRNA. **(D)** Colony formation assay for the assessment of cell survival in the groups treated as described above. **(E–G)** Transwell migration and invasion assays were performed in the groups treated as described above. *p < 0.05, **p < 0.01, ***p < 0.001.

Given that TAZ activation promotes cell survival, proliferation, and metastasis by stimulating its downstream transcription factors (Pocaterra et al., 2020), we next dissected the role of TAZ in the *H. pylori* infection-induced malignant transformation of gastric epithelial cells. Knockdown of endogenous TAZ by siRNA inhibited cell proliferation (Figures 3A,B, EDU assay) and cell viability (Figure 3C, CCK8 assay) induced by CagA+ *H. pylori* strain NCTC11637 or 7.13. compared with *H. pylori* infection alone in AGS cells. Similarly, the number of AGS cell clones was diminished after treatment with TAZ siRNA in combination with the *H. pylori* strain compared to *H. pylori* infection alone (Figure 3D). To explore how TAZ mediates cell invasion and migration induced by *H. pylori* infection, AGS cells were transfected with TAZ siRNA for 48 h and then cocultured with the *H. pylori* strain for a transwell assay. The siRNA-mediated knockdown of YAP in combination with CagA+ *H. pylori* NCTC11637 infection significantly decreased invasion and migration capacities (Figures 3E–G). These findings suggest that impaired TAZ activation abolishes *H. pylori* infection-induced malignant transformation including cell survival, proliferation, invasion and migration.

### *Helicobacter pylori* promoted the Wnt/β-catenin pathway through regulation of TAZ expression

**Figure 4:**
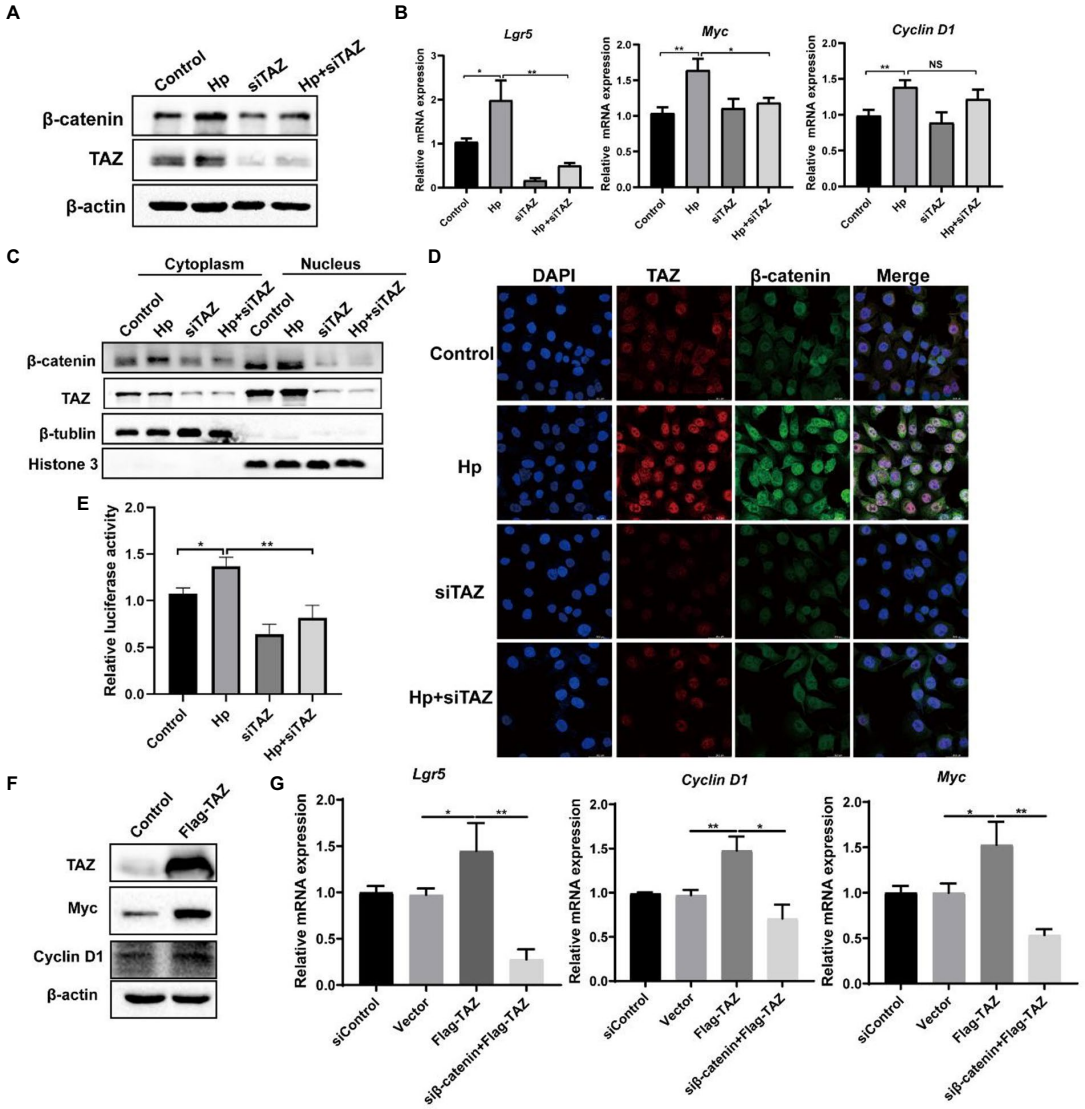
*Helicobacter pylori* infection promoted the β-catenin pathway *via* TAZ. **(A)** Western blot for total β-catenin and TAZ expression in gastric AGS cells transfected with TAZ siRNA and subsequently infected with *H. pylori*. **(B)** RT-PCR assay for mRNA levels of downstream genes of Wnt/β-catenin pathway in the groups treated as described above. **(C)** After transfection with TAZ siRNA and infection with *H. pylori*, cytoplasmic and nuclear protein fractions were isolated. Western blotting was performed to determine the protein levels of TAZ and beta-catenin. β-Tubulin and histone H3 served as loading controls for cytoplasmic and nuclear proteins, respectively. **(D)** Immunofluorescence staining for TAZ and β-catenin cellular localization in the groups treated as described above. **(E)** TOP-Flash luciferase reporter assay for β-catenin transcriptional activity in AGS cells transfected with TAZ siRNA and infected with the *H. pylori* strain. **(F)** Western blot analysis of the protein levels Myc and Cyclin D1, which are downstream effectors of β-catenin, in AGS cells transiently transfected with Flag-TAZ or empty vector. **(G)** RT–PCR analysis of the mRNA levels of downstream effectors of β-catenin, such as Lgr5, Cyclin D1 and Myc, in AGS cells transfected with Flag-TAZ alone or in combination with β-catenin siRNA. *H. pylori* strains in all experiments were used at an MOI of 100. *p < 0.05, **p < 0.01.

YAP/TAZ are closely linked to the Wnt/β-catenin pathway because they share some target genes and biological processes (Li et al., 2019). Given that our data indicated that *H. pylori* infection led TAZ overexpression and nuclear accumulation, we next investigated whether TAZ induction promotes β-catenin pathway activation in response to *H. pylori* infection. TAZ knockdown significantly downregulated the β-catenin protein levels induced by *H. pylori* (Figure 4A). In addition, qRT-PCR data showed that *H. pylori* infection upregulated the transcription of β-catenin-targeted genes including *Lgr5* and *Myc* which were then downregulated after treatment with TAZ siRNA (Figure 4B). Given that β-catenin activation is correlated with increased nuclear β-catenin abundance, we examined the effect of TAZ knockdown on the subcellular expression of β-catenin following *H. pylori* infection in AGS cells by separating the nuclear and cytoplasmic proteins. As shown in Figure 4C, *H. pylori* infection promoted nuclear TAZ and β-catenin compartmentalization, which was significantly suppressed by TAZ knockdown. Consistent with these findings, immunofluorescence assay showed that *H. pylori* infection-induced nuclear translocation was significantly suppressed by inhibition of TAZ activation (Figure 4D). Moreover, we performed a TOP/FOP flash luciferase reporter assay to detect β-catenin transcriptional activity. As shown in Figure 4E, *H. pylori* infection increased TCF/β-catenin reporter activity, and this effect was significantly inhibited by siRNA-mediated TAZ knockdown. To further confirm the effect of TAZ on the β-catenin pathway, AGS cells were transiently transfected with a TAZ overexpression plasmid. Exogenous TAZ overexpression increased the protein levels of Myc and Cyclin D1, the downstream targets of the Wnt/β-catenin pathway (Figure 4F). Consistent with these results, TAZ overexpression led to significant transcriptional induction of the downstream genes of Wnt signaling, such as *Lgr5*, *Cyclin D1*, and *Myc,* which was remarkably blocked following β-catenin knockdown by siRNA (Figure 4G). Collectively, these findings suggest that *H. pylori* infection leads to β-catenin activation and target gene expression through TAZ upregulation.

### TAZ activation in response to *Helicobacter pylori* infection was abrogated by β-catenin knockdown

**Figure 5:**
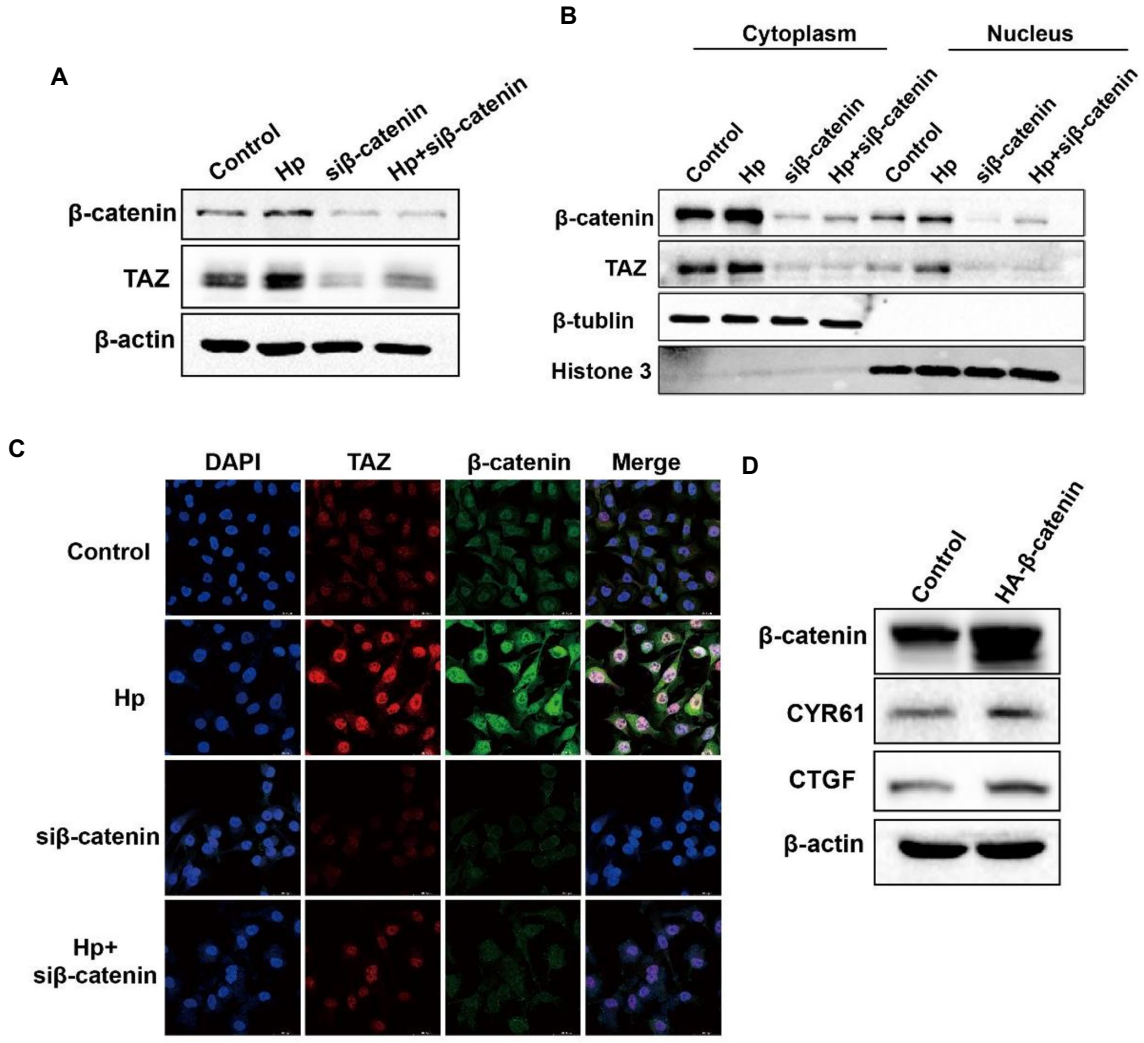
β-Catenin also acted as the upstream factor for TAZ activation and exhibited enhanced interaction with TAZ in response to *H. pylori* infection. **(A)** After knocking downβ-catenin by siRNA, AGS cells were cocultured with the *H. pylori* strain. Western blot analysis of TAZ total expression. **(B)** Western blot analysis of TAZ and β-catenin expression in the cytoplasmic and nuclear fractions after treatment with β-catenin siRNA and the *H. pylori* strain. β-Tubulin and histone H3 served as loading controls for cytoplasmic and nuclear proteins, respectively. **(C)** Immunofluorescence staining for TAZ and β-catenin subcellular localization in the groups treated as described above. **(D)** Western blot analysis of the protein levels of CTGF and CYR61 in AGS cells expressing HA-β-catenin plasmid or empty vector. *H. pylori* strains in all experiments were used at an MOI of 100.

We next explored the molecular mechanism of TAZ activation in response to *H. pylori* infection. Given the close relationship between the Hippo and Wnt signaling pathways, we hypothesized that *H. pylori* infection activates the TAZ pathway through the regulation of β-catenin. To test this hypothesis, gastric epithelial AGS cells were transfected with β-catenin siRNA and then cocultured with the *H. pylori* NCTC11637 strain. Knockdown of β-catenin significantly suppressed TAZ expression induced by *H. pylori* (Figure 5A). Moreover, cytoplasmic and nuclear proteins were effectively separated for the detection of the intracellular TAZ expression. Notably, *H. pylori* infection promoted the nuclear accumulation of TAZ and β-catenin, whereas β-catenin knockdown by siRNA remarkably inhibited this induction (Figure 5B). Immunofluorescence staining for TAZ and β-catenin confirmed that *H. pylori*-induced nuclear translocation of TAZ was inhibited by knockdown of β-catenin (Figure 5C). Furthermore, transient overexpression of β-catenin upregulated the protein levels of CTGF and CYR61 downstream of the Hippo signaling pathway (Figure 5D). Taken together, these findings suggest that a positive feedback loop exists between TAZ and the Wnt/β-catenin pathway in *H. pylori*-induced gastric tumorigenesis.

### CagA+ *Helicobacter pylori* infection enhanced the interaction of TAZ with β-catenin

**Figure 6:**
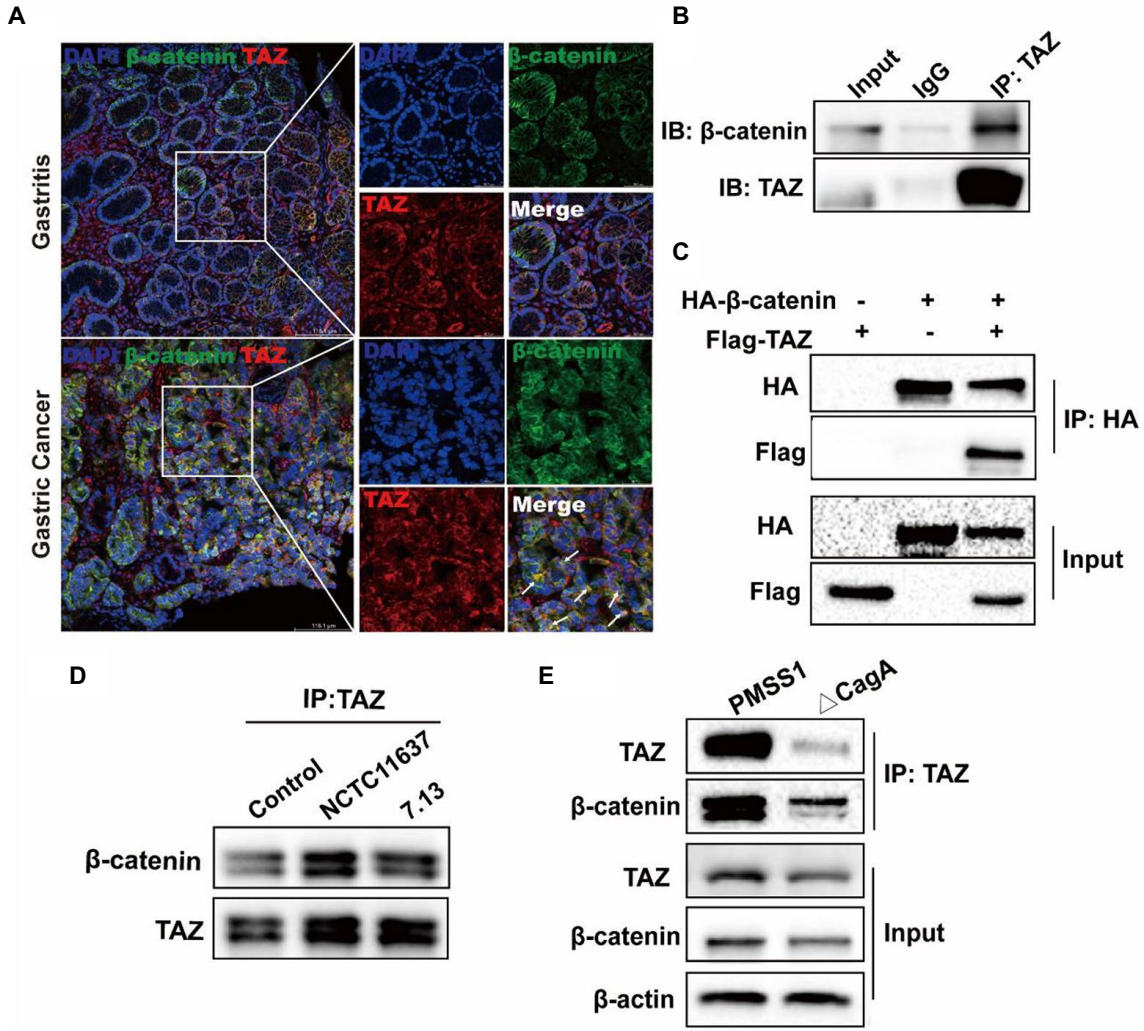
CagA+ *H. pylori* infection enhances the interaction of TAZ with β-catenin. **(A)** Immunofluorescence staining for TAZ and β-catenin colocalization in human gastritis and gastric cancer tissues (Magnification 200×, scale bars =116.1 μm; and digital zoom, scale bars = 38.7 μm). **(B)** Interaction between endogenous TAZ and β-catenin. Immunoprecipitation was performed with an anti-TAZ antibody followed by immunoblotting with the indicated antibodies. **(C)** Interaction between HA-β-catenin and Flag-TAZ. Immunoprecipitation was performed with an anti-HA antibody, followed by immunoblotting with the indicated antibodies. **(D)** AGS cells were cocultured with *H. pylori* NCTC11637 or 7.13 strain at an MOI of 100 for 6 h. Cell lysates were collected and then immunoprecipitated with anti-TAZ antibody. Western blotting was performed to assess TAZ and β-catenin expression. **(E)** After infection with the *H. pylori* wild-type strain or CagA- mutant for 6 h, cell lysates were collected. Infection with the *H. pylori* CagA- mutant resulted in a weak interaction of TAZ with β-catenin, compared with that treatment with wild-type *H. pylori* strain.

It has been reported that TAZ is associated with the destruction complex in the Wnt/β-catenin pathway, including Axin1, β-TrCP, β-catenin and GSK3β (Azzolin et al., 2014; Lee et al., 2020). Therefore, we delineated the possible interaction between TAZ and β-catenin in *H. pylori*-associated gastric carcinogenesis. The immunofluorescence assay was first performed to determine the expression and cellular localization of TAZ and β-Catenin in human gastric tissues. β-catenin was normally expressed in the membrane of epithelial cells of gastric mucosa with chronic gastritis, and TAZ was present in the plasma membrane and cytoplasm. In contrast, increased overall expression of gastric TAZ was observed, and TAZ colocalization with β-catenin was noted in human gastric cancer tissues but not gastritis tissues (Figure 6A). The interaction of endogenous TAZ with β-catenin was further demonstrated using the co-immunoprecipitation assay (Figure 6B). These data suggest that TAZ may be translocated from the plasma membrane and cytoplasm to the cellular nucleus, and directly interact with β-catenin in gastric tumorigenesis. Moreover, HA-tagged β-catenin was transiently coexpressed with Flag-tagged TAZ in gastric AGS cells, and the cell lysates were subjected to immunoprecipitation with an anti-HA antibody, demonstrating the strong exogenous interaction between HA-β-catenin and Flag-TAZ (Figure 6C). This interaction was significantly enhanced by coinfection with CagA+ *H. pylori* strain NCTC11637 or 7.13 (Figure 6D). Our data and recent studies have indicated that CagA is required for *H. pylori*-induced TAZ and β-catenin (Yong et al., 2016); w therefore, we investigated whether CagA is involved in the interaction between TAZ and β-catenin in response to *H. pylori* infection. Coimmunoprecipitation of TAZ with β-catenin in wild-type *H. pylori*-infected cells was stronger than that in CagA-deficient mutant strain-infected cells (Figure 6E). These data collectively indicated that *H. pylori* CagA plays an important role in the *H. pylori*-enhanced interaction between TAZ and β-catenin.

### Knockdown of β-catenin significantly suppressed TAZ-induced gastric cancer cell proliferation, migration, and invasion

**Figure 7:**
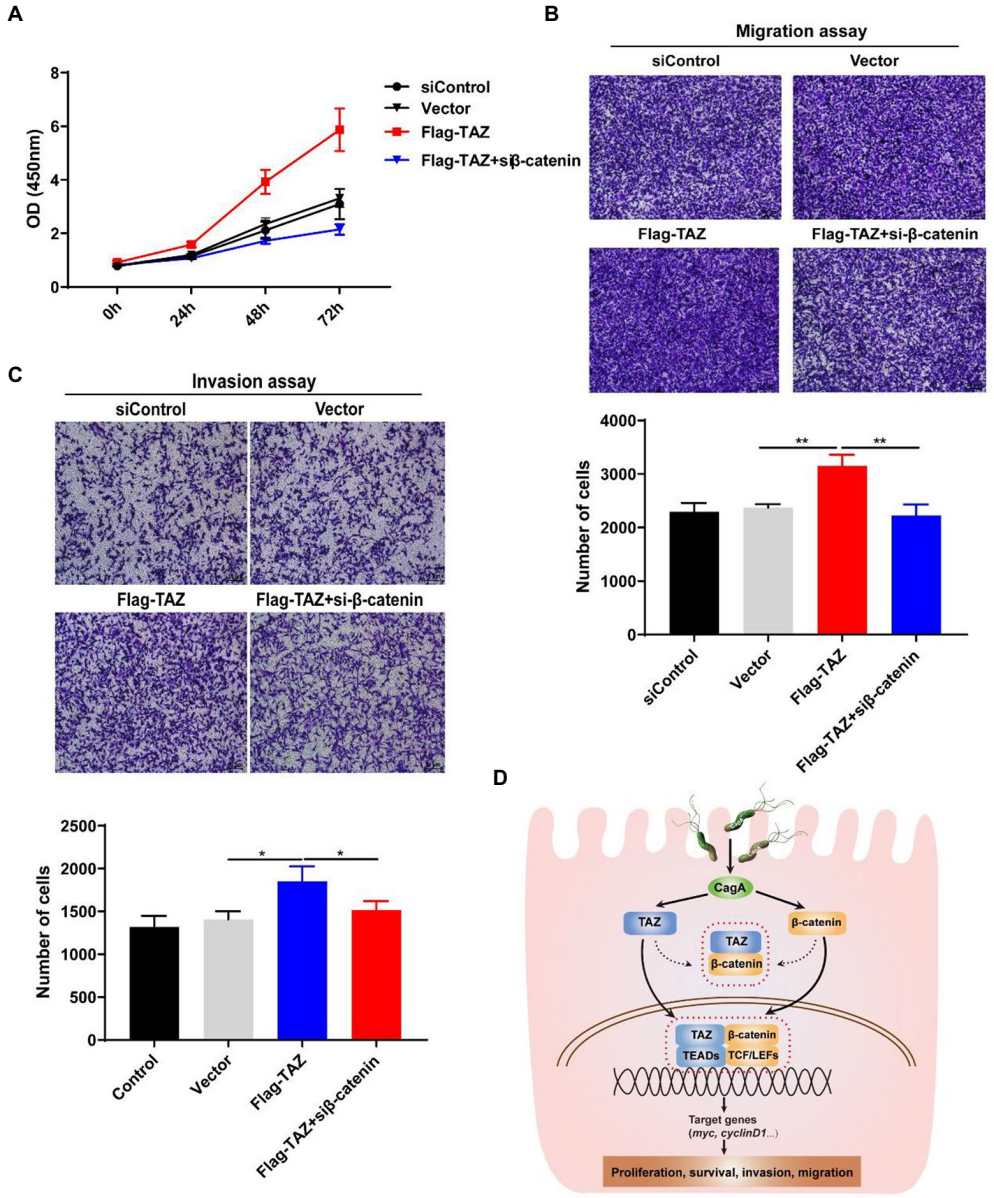
Knockdown of β-catenin significantly suppressed TAZ-induced cell proliferation, invasion, and migration. **(A)** CCK8 assay for cell proliferation in AGS cells treated as described for *A*. **(B,C)** Transwell assays for migration **(B)** and invasive **(C)** ability of AGS cells treated as described above. (Magnification 100×, scale bars =10 μm). **(D)** Schematic representation of the regulation of the TAZ/β-catenin axis by *H. pylori* infection in gastric carcinogenesis.

As our data indicated that *H. pylori* infection promotes the Wnt/β-catenin signaling pathway *via* the activation of TAZ, we next investigated whether β-catenin plays a role in regulating TAZ-mediated phenotypic alterations of gastric cancer cells. TAZ overexpression induced gastric cell proliferation, which was blocked by β-catenin siRNA (Figure 7A). Furthermore, β-catenin knockdown alleviated TAZ-induced gastric cancer cell migration and invasion (Figures 7B,C). Taken together, knockdown of β-catenin suppressed TAZ-mediated cell proliferation, migration and invasion.

## Discussion

*H. pylori* is generally acquired during childhood and remains in the stomach for a lifetime if untreated (Suerbaum and Michetti, 2002; Crowe, 2019). It has been documented that gastrointestinal diseases, particularly gastric adenocarcinoma, are closely associated with *H. pylori* infection (Crowe, 2019). The prevalence of *H. pylori* infection varies greatly among geographic regions. Notably, East Asian countries have a high incidence of gastric carcinoma, which is mainly attributed to the high prevalence of *H. pylori* infection (Inoue, 2017; Wong et al., 2021). At present, the molecular mechanisms underlying *H. pylori* infection-induced gastric carcinogenesis are not completely understood. We previously reported that *H. pylori* infection induces the epithelial-mesenchymal transition and contributes to gastric malignant transformation *via* activation of the YAP pathway (Li et al., 2018). We reported increased TAZ expression in human gastric cancer tissues compared with chronic gastritis tissues as well as *H. pylori*-positive gastritis patients compared with *H. pylori*-negative patients. In addition, *H. pylori* infection significantly elevated TAZ expression and its nuclear translocation in a CagA-dependent manner both in the INS-GAS mouse model and cultured human gastric cells. In addition, our results indicated that TAZ is required for *H. pylori-*induced activation of the Wnt/β-catenin pathway, and the reverse is true for β-catenin. These effects likely occur *via* their direct interaction. Furthermore, *H. pylori* CagA plays an important role in enhancing the interaction between TAZ and β-catenin. Finally, we showed that the interactive regulation of activation between TAZ and β-catenin is integral in *H. pylori* infection-associated gastric epithelial cell malignant transformation as demonstrated by increased cell proliferation, invasion and migration. Taken together, our data reveal a positive feedback loop mechanism between the YAP orthologue TAZ and the β-catenin pathway in *H. pylori* infection-induced gastric tumorigenesis (Figure 7D), providing new insights into the mechanism of *H. pylori* pathogenicity.

As core effectors of the Hippo pathway, TAZ and its related protein YAP have been widely characterized in the regulation of cell growth, tissue regeneration and organ size (Mohajan et al., 2021). Although TAZ and YAP share similarities in amino acid sequences, TAZ can be clearly distinguished based on its structure, function and regulatory network (Jeong et al., 2021; Reggiani et al., 2021). Our previous studies have identified the role of YAP in the pathogenesis of *H. pylori* infection. Supporting this role, *Molina-Catro* et al. also demonstrated the relationship between *H. pylori* and YAP (Molina-Castro et al., 2020). Subsequently, this research team found that *H. pylori* increased TAZ expression and nuclear accumulation. Additionally, TAZ was overexpressed in human gastric cancer tissues (Tiffon et al., 2020). We further indicated that the increase in TAZ expression seems to be dependent of *H. pylori* infection in gastritis tissues rather than gastric cancer tissues. A significant induction of TAZ expression was also observed in *H. pylori*-infected mice, which developed gastric inflammation after infection. This result further supports the role of *H. pylori* as the initiation factor in precancerous lesions. A lower abundance of *H. pylori* was previously observed in human gastric cancer (Ozbey et al., 2020). This study showed for the first time that *H. pylori* infection-induced TAZ activation was dependent on the virulence factor CagA. Furthermore, TAZ activation is required for *H. pylori* infection-induced gastric cell proliferation, invasion, and migration, as demonstrated by CCK8, EdU, colony formation, and transwell assays. Notably, our data showed that *H. pylori*-positive gastritis tissues had higher TAZ levels than *H. pylori*-negative tissues. Therefore, we hypothesized that TAZ upregulation may occur in the early stages of *H. pylori* infection.

Given the overlapping roles in several biological functions, recent studies have explored the molecular interplay between the YAP/TAZ and β-catenin pathways. Tripath et al. indicated that TAZ directly inhibited β-catenin transcriptional activity in muscle cells and further affected skeletal muscle differentiation (Tripathi et al., 2022). TAZ exhibited entirely different regulatory effects on β-catenin in other diseases. *Lee* et al. clarified the regulatory mechanism of TAZ on the Wnt/β-catenin signaling pathway in ADPKD caused by genetic mutation of PKD1 or PKD2. TAZ strongly interacts with AXIN1, the core component of destruction complex, thereby increasing β-catenin activity and downstream c-MYC expression (Lee et al., 2020). This study innovatively revealed the crosstalk between TAZ and Wnt/β-catenin in *H. pylori*-associated gastric carcinogenesis. Our experiments first indicated that TAZ knockdown significantly inhibited the expression, nuclear translocation and transcriptional activity of β-catenin in response to *H. pylori* infection. Additionally, β-catenin knockdown suppressed *H. pylori*-induced TAZ expression. These data suggest a positive feedback loop between TAZ and β-catenin in the pathogenesis of *H. pylori* infection. Consistent with these findings, some evidence supports the synergistic effect of YAP/TAZ and the Wnt/β-catenin signaling pathway. TAZ acts in concert with β-catenin to promote hepatoblastoma development (Zhang et al., 2020). YAP and TAZ are transcriptionally activated upon β-catenin activation, thereby promoting liver tumorigenesis (Bisso et al., 2020). Silencing of the Hippo upstream kinases Mst1 and Mst2 could activate the activity of YAP/TAZ and Wnt/β-catenin signaling, resulting in rapid hepatocellular carcinoma formation. Additionally, the positive feedback loop between Notch signaling and YAP/TAZ could be inhibited by the Wnt/β-catenin pathway (Kim et al., 2017).

Some evidence in support of the relationship between the other components of Hippo signaling and the Wnt/β-catenin pathway has been reported. Wnt3a and Wnt5a were identified as potent activators of YAP/TAZ (Park et al., 2015). The Wnt scaffolding protein DVL interacts with YAP in a phosphorylation-dependent manner (Lee et al., 2018). A recent study reported that YAP/TAZ physically interacts with β-catenin. In the “Wnt-off” state, YAP/TAZ could be sequestered in the β-catenin destruction complex and associated with Axin1, β-catenin, GSK3, and β-TrCP (Azzolin et al., 2014). Likewise, our observation suggested that both endogenous and exogenous TAZ interacted with β-catenin. Furthermore, infection with two *H. pylori* strains, NCTC11637 and 7.13, enhanced the interaction between TAZ and β-catenin. Intriguingly, we found that CagA plays an important role in their interaction by employing the PMSS1 strain and its CagA- isogenic mutant. VacA, another important virulence factor, is responsible for *H. pylori*-induced cellular vacuolation and gastric injury (Chauhan et al., 2019). Further studies will investigate the role of VacA in the interaction between TAZ and β-catenin by constructing an *H. pylori* VacA- isogenic mutant. In summary, these findings also indicated that β-catenin was responsible for ectopic TAZ-induced gastric cell proliferation, migration and invasion. Based on our findings, we established a regulatory feedback loop underlying the relationship between TAZ and β-catenin, which promotes *H. pylori* infection-induced gastric tumorigenesis.

## Conclusion

In summary, our data demonstrated that *H. pylori* infection triggers gastric epithelial cell malignant transformation *via* the promotion of TAZ activation. Mechanistically, *H. pylori* infection leads toβ-catenin pathway activation *via* TAZ, which contributes to gastric carcinogenesis. In turn, β-catenin functions as an upstream regulator and is involved in TAZ activation following *H. pylori* infection. Moreover, we implicated the important role of CagA in TAZ regulation by *H. pylori*. Studies further confirmed the clinical evidence that *H. pylori* positive gastritis tissues contain higher TAZ expression levels than *H. pylori*-negative tissues. This study effectively linked *H. pylori* infection and the bacterial protein CagA to TAZ and the β-catenin pathway, thereby elucidating a new pathogenic mechanism of *H. pylori* and suggesting novel targets for the prevention and early detection of gastric cancer.

## Data availability statement

The original contributions presented in the study are included in the article/Supplementary material, further inquiries can be directed to the corresponding authors.

## Ethics statement

The studies involving human participants were reviewed and approved by the Ethics Committees of The First Affiliated Hospital of Nanchang University. The patients/participants provided their written informed consent to participate in this study. The animal study was reviewed and approved by the ethics committees of The First Affiliated Hospital of Nanchang University.

## Author contributions

XX, NLi, and YZh: contributed to the design of this study, performance of the experiments, and analysis of data. CS, XW, YO, HW, and YZho: conducted various portions of the experiments. XH, CH, and NLu: analyzed immunohistochemical data. NLi, XX, and ZG: drafted the manuscript. CX, CH, JH, NLu, YZh, and NLi: contributed to the study supervision and coordination. NLi, CH, and YZh: obtained funding support. All the authors critically revised the manuscript and provided intellectual content.

## Funding

This work was supported by the National Natural Science Foundation of China (81900500, 81870395, and 82170580), Natural Science Foundation of Jiangxi Province (20212BAB216016), Doctoral Research Initiation Funding (701221002), and Young Medical Teacher Training Fund of Nanchang University (4209-16100009-PY201923).

## Conflict of interest

The authors declare that the research was conducted in the absence of any commercial or financial relationships that could be construed as a potential conflict of interest.

## Publisher’s note

All claims expressed in this article are solely those of the authors and do not necessarily represent those of their affiliated organizations, or those of the publisher, the editors and the reviewers. Any product that may be evaluated in this article, or claim that may be made by its manufacturer, is not guaranteed or endorsed by the publisher.

## Abbreviations

*H. pylori*, *Helicobacter pylori*
CagA, Cytotoxin-associated gene A
PPI, proton pump inhibitor
YAP, Yes-associated protein
TAZ, PDZ-binding motif
EMT, epithelial-mesenchymal transition
INS-GAS, Insulin–gastrin
FBS, fetal bovine serum
BSA, bovine serum albumin
CCK-8, Cell counting kit-8
SD, standard deviation
ADPKD, autosomal-dominant polycystic kidney disease
DVL, Disheveled
